# Coverage of Community-Based Management of Severe Acute Malnutrition Programmes in Twenty-One Countries, 2012-2013

**DOI:** 10.1371/journal.pone.0128666

**Published:** 2015-06-04

**Authors:** Eleanor Rogers, Mark Myatt, Sophie Woodhead, Saul Guerrero, Jose Luis Alvarez

**Affiliations:** 1 Coverage Monitoring Network (CMN) at Action Against Hunger UK, First Floor, Rear Premises, London, United Kingdom; 2 Brixton Health, Alltgoch Uchaf, Llawr-y-glyn, Powys, United Kingdom; University of Alabama at Birmingham, UNITED STATES

## Abstract

**Objective:**

This paper reviews coverage data from programmes treating severe acute malnutrition (SAM) collected between July 2012 and June 2013.

**Design:**

This is a descriptive study of coverage levels and barriers to coverage collected by coverage assessments of community-based SAM treatment programmes in 21 countries that were supported by the Coverage Monitoring Network. Data from 44 coverage assessments are reviewed.

**Setting:**

These assessments analyse malnourished populations from 6 to 59 months old to understand the accessibility and coverage of services for treatment of acute malnutrition. The majority of assessments are from sub-Saharan Africa.

**Results:**

Most of the programmes (33 of 44) failed to meet context-specific internationally agreed minimum standards for coverage. The mean level of estimated coverage achieved by the programmes in this analysis was 38.3%. The most frequently reported barriers to access were lack of awareness of malnutrition, lack of awareness of the programme, high opportunity costs, inter-programme interface problems, and previous rejection.

**Conclusions:**

This study shows that coverage of CMAM is lower than previous analyses of early CTC programmes; therefore reducing programme impact. Barriers to access need to be addressed in order to start improving coverage by paying greater attention to certain activities such as community sensitisation. As barriers are interconnected focusing on specific activities, such as decentralising services to satellite sites, is likely to increase significantly utilisation of nutrition services. Programmes need to ensure that barriers are continuously monitored to ensure timely removal and increased coverage.

## Introduction

The importance of high coverage of public health interventions is well recognised [1]. The product of coverage and effectiveness defines the impact of an intervention, [2, 3] an essential indicator of programme success. Prior to the current model, which treats severely malnourished children in the community, services were provided in Therapeutic Feeding Centres (TFCs). These in-patient units, set up specifically to treat severely malnourished children, were often centrally based in towns requiring most families to travel long distances to reach them as well as demanding long stays for both child and carer. Although they cured a high proportion of children that attended, implementers found that the costs to families associated with receiving treatment were too high, both in terms of financial and opportunity costs, so few were attending. The cost of transport to the centre, multiple days spent away from work and other duties and having to leave siblings at home were all barriers to access to care, leading to low coverage [4–6]. TFC coverage was also limited by bed availability, with each centre typically treating fewer than 30 cases at any one time with a treatment episode lasting between five and eight weeks. Unsurprisingly TFCs served only between 4% and 10% of the affected population [7, 8]. A belief that coverage could be improved through adaptations to services, combined with the development of ready-to-use food (RUTF), led to the creation of the Community-based Management of Acute Malnutrition (CMAM) model used today.

Community-based treatment of severe acute malnutrition (SAM) allows the majority of malnourished children to be treated at an outpatient clinic on a weekly or fortnightly basis with only the most severe cases being admitted to inpatient centres for short periods. The outpatient clinic monitors the child’s response to the treatment and provides antimicrobial, antihelminthic, and antimalarial drugs; vitamin A supplementation; and measles vaccination (if required) before sending them home with sufficient ready to use therapeutic food (RUTF) to last until the following visit, allowing recovery to take place in the community [8]. This model has been deemed a success, performing consistently well in a variety of contexts, achieving high cure rates (> 90%), low death rates (< 2%), and low default (< 10%) rates [9]. It is now widely implemented as part of routine government services and in humanitarian emergencies across Africa, Asia and the Americas [10].

Initial coverage assessments carried out during the development of the CMAM model showed that high levels of coverage (e.g. > 70%) were achievable and a significant improvement on what had been attained by the in-patient model [11]. However, since the scale up of the community-based approach, coverage data has been limited with only a few programme implementers measuring coverage. This is largely due to a poor understanding of the role of coverage in delivering programme impact but also because rapid and low cost tools to assess coverage have only recently become available. With the development and adoption of these reliable coverage assessment methods, practices are beginning to change [12]. The standard tools used are the Semi-Quantitative Evaluation of Access and Coverage (SQUEAC) and Simplified Lot Quality Assurance Sampling Evaluation of Access and Coverage (SLEAC) methods [12]. Both methods are very similar in the tools they employ and their main difference lays in the aim and the scale of the investigation that is conducted: SQUEAC is adapted to small areas and gives a detailed analysis of programme barriers (also known as “bottlenecks”) and boosters to access services while SLEAC can be implemented in wider areas and focuses on coverage estimates [12]. Periodic coverage assessments can feed in to an audit cycle allowing continued improvements with the aim of achieving and sustaining best practice over time [3, 12].

The Coverage Monitoring Network (CMN) has collected and collated data from over 40 of these coverage assessments to build an evidence base and make conclusions about the impact of community-based SAM treatment, which has not been possible up until this point [13].

## Material and Methods

### Study design

This is a descriptive study of data on coverage and barriers to coverage collected by assessments of Community Management of Acute Malnutrition (CMAM) programmes in twenty-one countries supported by the Coverage Monitoring Network between July 2012 and June 2013. Forty-three SQUEAC assessments and one SLEAC assessment were carried out by CMN’s coverage advisors, or those they had trained, and had reports available at the time of this review. The majority of assessments were carried out in Africa. The remaining five assessments were implemented in Afghanistan, Haiti, Nepal, Pakistan and the Philippines.

### Data and statistical methods

Assessments took place in SAM treatment programmes which contacted the CMN for support. They were predominantly (*n* = 34) undertaken in programmes implemented by local Ministries of Health (MoH) with varying degrees of NGO support. Ten assessments were undertaken in programmes directly implemented by NGOs. Programmes that were assessed included those implemented in rural and urban settings and refugee and internally displaced persons camps.

In this analysis, the barriers to access are those cited by principal carers of SAM children not enrolled in a SAM treatment programme and found in wide-area surveys using active and adaptive or door-to-door case-finding [12]. Only the primary barrier to access cited by the carer is included in assessments and the most frequently reported barrier was identified from each assessment for the analysis reported here. The reported barriers are listed in Table 1. Most of the barriers are self-explanatory although three may require further clarification to ensure their appropriate interpretation by the reader:

- “High opportunity costs” describes the decision made by carers not to attend treatment services because the direct and indirect costs and the implications of attending (e.g. travel costs, loss of income, loss of agricultural labour, etc.) are perceived as being too high.
- “Inter-programme interface problems” describes a child with SAM that seeks treatment or is already enrolled in a related treatment service at a health facility (e.g. malaria treatment) but is not identified as being eligible for SAM treatment and is not, therefore, transferred or referred for SAM treatment.
- “Previous rejection” describes a child with SAM that has previously attended the programme for screening or had been referred to treatment by programme staff or from another programme but was subsequently found not to meet the programme’s admission criteria and thus did not receive treatment at that time. Carers are then less willing to follow-up future referrals fearing further rejection [14].

**Table 1:** Categories of barrier used in the analysis.

Class of barrier	Specific barrier
**Failures of community sensitisation and mobilisation**	Lack of awareness about malnutrition (signs, aetiology, treatment)
	Lack of awareness about the CMAM programme
	Husband refused to allow the child to attend the CMAM programme
	Stigma / shame
	Traditional health practitioners preferred to CMAM programme
**At-clinic failures**	No ready-to-use-therapeutic food (RUTF) at clinic
	Inter-programme interface problems
	Wrong admission and discharge criteria
	Poor delivery of service (staff abusive, demand money, staff absent)
	Previous rejection of a child known to the carer
	Previous rejection of the current SAM case
	Long waiting times at clinic
	Defaulted due to non-response
	Relapse but not returned to the programme
**Problems with locations of CMAM sites**	Carer busy / high opportunity costs
	Lack of money to pay for transport
	Distance from home to CMAM site
**Other barriers**	Insecurity (e.g. banditry, abduction, rape gangs)
	Seasonal barriers (e.g. high rainfall, high temperatures)
	Population movement (e.g. transhumance)
	Carer sick
	Other barriers (not otherwise specified)

The levels of coverage reported in each assessment was compared against “SPHERE Project” minimum standards [15] which are an internationally recognised set of common principles and universal minimum standards in life-saving areas of humanitarian response. There are three different thresholds based on the location setting as follows; 50% for rural settings, 70% for urban settings and 90% for camp settings.

### Ethics

The current study did not go through an Ethical Review Board. This paper is not an active research study but a collation of different assessments implemented by the CMN. Each of those assessments followed appropriate ethical processes and sought written or verbal informed consent from all participants. Different methods were followed in each country due to the different contexts. For this analysis, no further consent was sought since it was a retrospective analysis of previously collected data. Only de-identified aggregate (population-level) data were used in this study.

## Results

**Fig 1:**
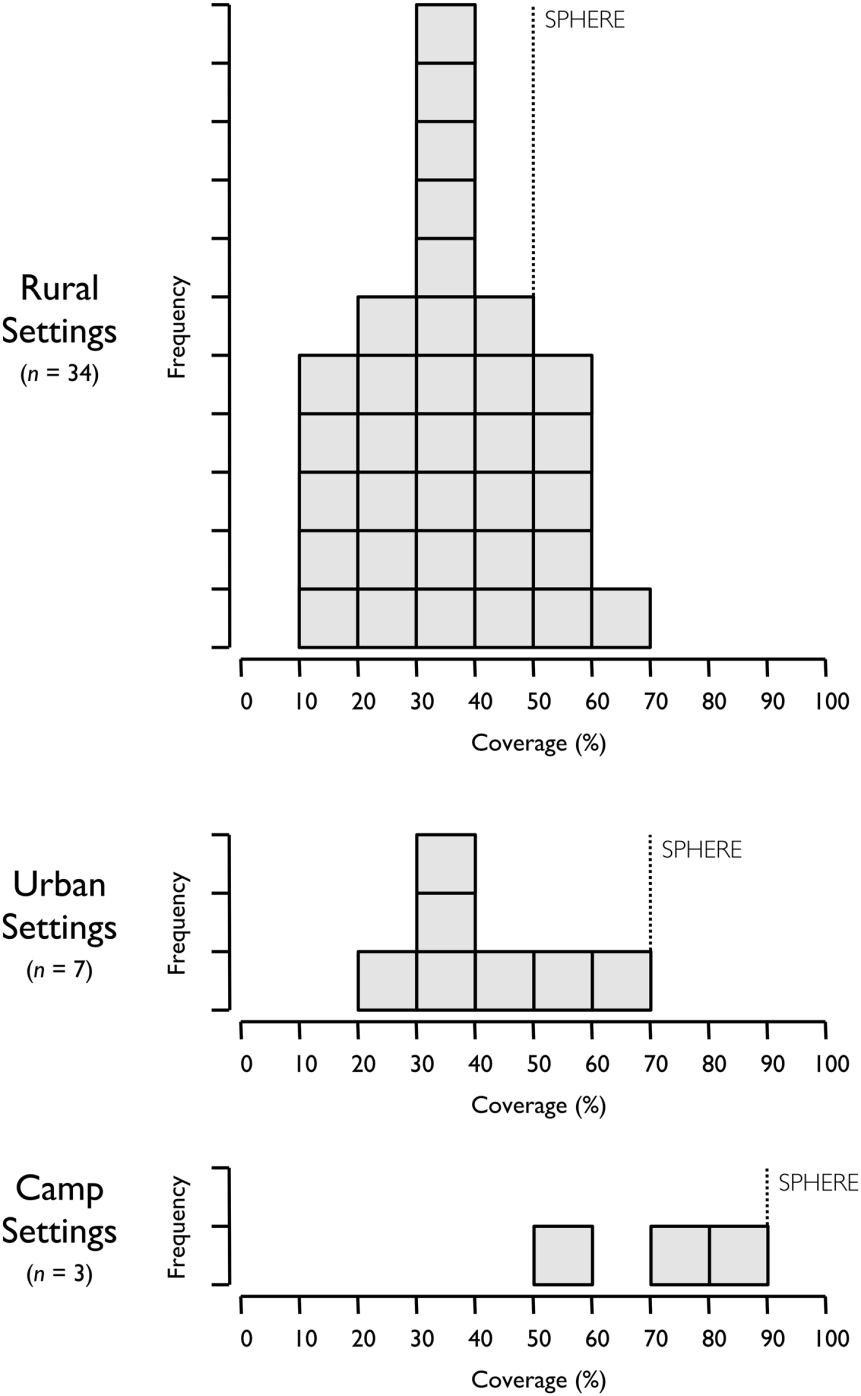
Distribution of coverage estimates from 44 coverage assessments.

The distributions of coverage from the 44 assessments are presented in Fig 1. Context-specific SPHERE standards are marked by an upright dotted line.

The majority of coverage estimates (i.e. 38 of 44) did not reach the minimum coverage standards set by the SPHERE project (50%, 70% and 90% for rural, urban, and camp settings respectively). The average (mean) level of estimated coverage achieved by the programmes in this analysis was 38.3%. For rural programmes, it was 34.6%, for urban 40.9% and for camp settings, 74.2%.

**Fig 2:**
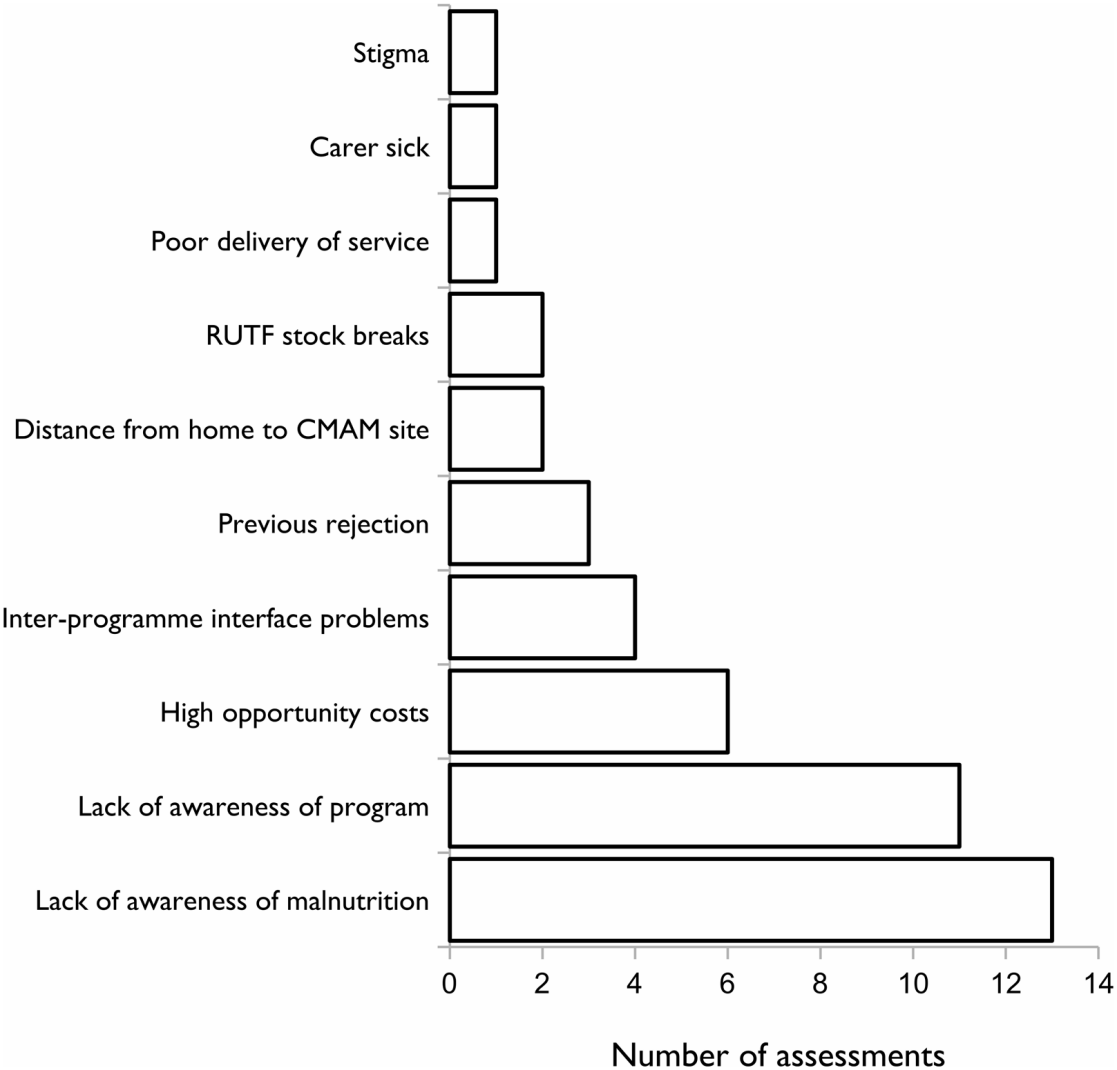
Primary barrier to access reported in each assessment by carers of non-covered SAM cases.

The most frequently cited barriers to access are presented in Fig 2.

Ranked by frequency, the five most commonly reported barriers to access across all programmes were lack of awareness of malnutrition, lack of awareness of the programme, high opportunity costs, inter-programme interface problems, and previous rejection. These five barriers account for 84% of all barriers reported. The first two barriers (i.e. lack of awareness of malnutrition and lack of awareness of the programme) account for just over half (55%) of all reasons given for non-attendance.

The barriers reported by programmes which reached SPHERE standards were compared with those found in programmes that did not. This is shown in Table 2. Only six programmes reached SPHERE standards, reporting four barriers to access: a lack of awareness about malnutrition and the programme, previous rejection of the current SAM case and high opportunity costs. All of these barriers were also reported in programmes which did not reach SPHERE standards.

**Table 2:** Primary barriers found in programmes that did and did not reach SPHERE standards.

	Programmes that did not reach SPHERE standards		Programmes that reached SPHERE standards	
Barriers	Frequency	%	Frequency	%
Lack of awareness about malnutrition (signs, aetiology, treatment)	11	28.9%	2	33.3%
Lack of awareness about the CMAM programme	9	23.7%	2	33.3%
Husband refused to allow the child to attend the CMAM programme	0		0	
Stigma / shame	1	2.6%	0	
Traditional health practitioners preferred to CMAM programme	0		0	
No ready-to-use-therapeutic food (RUTF) at clinic	2	5.3%	0	
Inter-programme interface problems	4	10.5%	0	
Wrong admission and discharge criteria	0		0	
Poor delivery of service (staff abusive, demand money, staff absent)	1	2.6%	0	
Previous rejection of a child known to the carer	0		0	
Previous rejection of the current SAM case	2	5.3%	1	16.7%
Long waiting times at clinic	0		0	
Defaulted due to non-response	0		0	
Relapse but not returned to the programme	0		0	
Carer busy / high opportunity costs	5	13.2%	1	16.7%
Lack of money to pay for transport	0		0	
Distance from home to CMAM site	2	5.3%	0	
Insecurity (e.g. banditry, abduction, rape gangs)	0		0	
Seasonal barriers (e.g. high rainfall, high temperatures)	0		0	
Population movement (e.g. transhumance)	0		0	
Carer sick	1	2.6%	0	
Other barriers (not otherwise specified)	0		0	
**Total:**	**38**	**100.0%**	**6**	**100.0%**

## Discussion

The analysis presented here shows that coverage of current programmes is lower than that achieved by the early version of the community based model (Community-based Therapeutic Care, CTC) research and development programmes, implemented by the model’s creators [16]. This indicates that impact will also be lower as low coverage and effectiveness are the main components of programme impact [2]. This is demonstrated through a comparison of the data: nine early CTC programmes from rural settings reported coverage levels ranging between 56% and 82% with an average of 72.5% [16]. Yet data from rural programmes reviewed in this analysis found the average (mean) level of coverage to be just under half of that. Although the resources directed towards the early CTC programmes means they may not be directly comparable with programmes in the current global scale-up of community-based SAM treatment, they do represent the potential of a community-based delivery model to achieve coverage levels above SPHERE minimum standards. Many programmes are failing to achieve even the lowest SPHERE minimum standard (i.e. 50%). This suggests that the current service delivery models, when operating at scale, are unable to provide the level of access required by the communities they serve and replication of the success of early research programmes is proving challenging in operational contexts. As there is an ongoing and necessary global scale up of SAM treatment services, [17] identifying solutions to the barriers to access in the model is essential.

There are limitations to the data. First, although this is the largest database of SAM treatment coverage assessments from operational settings, it remains small, limiting deeper analysis of specific sub-groups such as programme settings and programme delivery models. Second, as programmes were required to contact the CMN to receive support in undertaking assessments there may be selection bias towards favouring higher performing programmes and programmes supported by CMN members. Third, only the primary barrier reported by carers is included in the assessment. As multiple barriers are likely to result in the absence of treatment, this limits the depth of the analysis, including a multi-factor analysis. Fourth, whether there is a difference in importance between those barriers that are found in a programme that achieves SPHERE and those found in programmes that fail to reach SPHERE has not been explored, so they have been given equal weighting in this analysis which may bias certain barriers.

### Barriers to access

Although it is not possible to say whether these barriers to access cause low coverage, their removal will help eliminate their role in this outcome. This analysis emphasises the role programme implementers should play in strengthening aspects of treatment services such as community sensitisation / mobilisation, site selection, and case-finding / referral by community-based health workers. Previous published data from twelve coverage surveys on early CTC programmes across five African countries reported similar barriers to access supporting the findings of this analysis, although the factor deemed most significant varies [14]. Previous rejection from the programme was the most commonly reported factor in the previous analysis but lack of awareness of the programme and malnutrition were more commonly reported in this analysis. Opportunity costs and distance to site were significant for both. The persistence of the five most frequently reported barriers shows programmes in all contexts are facing similar issues emphasising the importance of sharing lessons learned [18].

### Community Sensitisation

The persistence of lack of awareness as a barrier suggests that effective engagement with beneficiary communities is often lacking. Previously published barrier analysis and coverage surveys supports this analysis showing a lack of awareness of the programme to be the second most common reason for non-attendance [14, 19, 20]. Additionally, of the six programmes which reached SPHERE in this analysis, a lack of awareness about the programme or malnutrition was reported as the main barrier for four of them, suggesting that strengthening community sensitisation activities remains a key activity to increase coverage even in more successful programmes.

Engagement is particularly challenging in resource-limited settings where health systems can be weak and with limited reach to the population. However, often the outreach element of the CMAM model is not prioritised leading to the dominant model of delivery being facility based and relying on passive case-finding—cases that are found by health workers when the child attends for another reason—and recruitment. A continuous dialogue is necessary between the programme and the community which can be led by outreach worker networks, which when linked to the health system, are one of the few ways this issue has been addressed effectively [21]. The simple activities outlined in the 2010 analysis, such as developing context appropriate messages based on socio-cultural assessments and using local channels of communication, remain critical to increase community awareness [14].

### Opportunity Costs and Distance

High opportunity costs associated with receiving care and distance to service delivery units (e.g. health centres and posts) are interconnected issues that have a significant impact on SAM treatment uptake. Both barriers have been consistently reported in the early CTC and more recent CMAM programmes [14]. Opportunity costs have been reduced with the transition from inpatient to outpatient care, and the decentralisation of care away from Therapeutic Feeding Centres (TFCs) in secondary or tertiary health facilities to primary healthcare facilities [11] but these barriers continue to limit access. Context-specific measures are required to address these barriers if community-based SAM treatment is to take steps towards being universally accessible. Identifying ‘acceptable’ distances and opportunity costs is complex as perceptions vary depending on the context and the season [18]. Common approaches include the use of mobile clinics or decentralising services to satellite sites. Further decentralisation, with community health workers delivering services door to door, has also shown success in achieving high coverage rates [21].

### Effects of barriers to access

**Fig 3:**
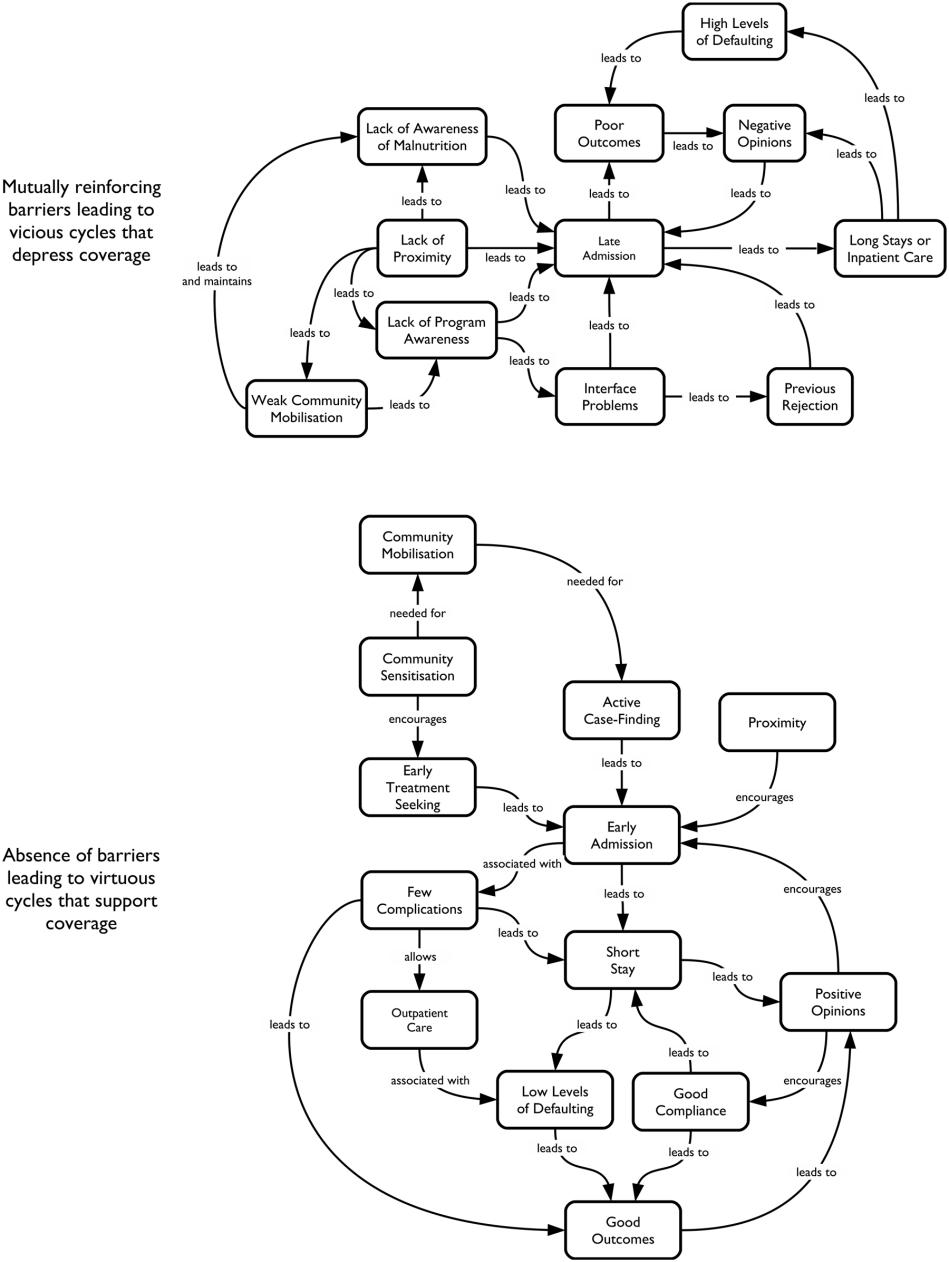
Mutually reinforcing barriers and boosters to coverage and effectiveness. These two concept maps are composites of concept maps from several SQUEAC coverage assessments with programme-specific details removed and using a standardised terminology. The starting points for the maps were SQUEAC coverage assessments from CMAM programmes in Sierra Leone and Bangladesh [12, 20,24].

Experience has shown that the effects of these access failures are threefold: Limited compliance with treatment, high rates of defaulting from the programme, and increased severity of disease at admission [22]. Programmes with these characteristics will have a limited impact because they will have low cure rates. Combined with low coverage, programme effectiveness is minimal. These factors are mutually reinforcing. Breaking the negative cycle can lead to good clinical outcomes and positively affect coverage (see Fig 3). Ineffective programmes with low coverage are also less cost-effective as the delayed case finding and subsequent long treatment times and negative outcomes increase the cost per DALY averted [23]. Therefore an additional gain made in actively addressing these barriers to access is that programmes will become more cost-effective as was demonstrated in the move from TFCs to outpatient treatment [23].

## Conclusions

This study shows that coverage of CMAM is lower than previous analyses of early CTC programmes; therefore reducing programme impact. Barriers to access need to be addressed in order to start improving coverage by paying greater attention to certain activities such as community sensitisation. As barriers are interconnected focusing on specific activities, such as decentralising services to satellite sites, is likely to increase significantly utilisation of nutrition services. Programmes need to ensure that barriers are continuously monitored to ensure timely removal and increased coverage.

## References

[1] Victora CG, Hanson K, Bryce J, Vaughan JP. Achieving universal coverage with health interventions. Lancet. 2004; 364 (9444):1541–8. 15500901 10.1016/S0140-6736(04)17279-6

[2] Habicht JP, Victora CG, Vaughan J P. Evaluation designs for adequacy, plausibility and probability of public health programme performance and impact. Int. J. Epidemiol. 1999; 28, 10–18. 10195658 10.1093/ije/28.1.10

[3] Tanahashi T. Health service coverage and its evaluation. Bull World Health Organ. 1978; 56: 295–303. 96953 PMC2395571

[4] Guerrero S, Gallagher M. Quantity through quality: scaling up CMAM by improving programme access. 2013. Available: http://www.ennonline.net/pool/files/fex/fx-44-web-(1).pdf

[5] Collins S. Paper 48: Community-based Therapeutic Care. Humanitarian Practice Network. 2004.

[6] Van Damme W, Boelaert M., Correspondence. The Lancet. 2002; 359(9302): 260–1. 11812590 10.1016/s0140-6736(02)07422-6

[7] Sadler K. Community-based Therapeutic Care: treating severe acute malnutrition in sub-Saharan Africa. PhD Thesis: Institute of Child Health, University College London. 2008. Available: http://discovery.ucl.ac.uk/16480/1/16480.pdf.

[8] Collins S, Dent N, Binns P, Bahwere P, Sadler K, Hallam A. Management of severe acute malnutrition in children. Lancet. 2006; 368(9551): 1992–2000. 17141707 10.1016/S0140-6736(06)69443-9

[9] Guerrero S, Rogers E. Access for All, Volume 1: Is community-based treatment of severe acute malnutrition (SAM) at scale capable of meeting global needs? 2013 Coverage Monitoring Network, London Available: http://www.coverage-monitoring.org/wp-content/uploads/2013/07/AAH-Policy-Paper-Comp.pdf

[10] UNICEF. Global SAM management update: Summary of findings. UNICEF, New York 2013 Available at http://reliefweb.int/sites/reliefweb.int/files/resources/Global%20SAM%20Management%20Update.pdf

[11] Sadler K, Myatt M, Feleke T, Collins S. A comparison of the programme coverage of two therapeutic feeding interventions implemented in neighbouring districts of Malawi, Public Health Nutrition. 2007; 10(9):907–913 17466097 10.1017/S1368980007711035

[12] Myatt M, Guevarra E, Fieschi L, Norrison A, Guerrero S, Schofield L *et al* Semi-Quantitative Evaluation of Access and Coverage (SQUEAC) / Simplified Lot Quality Assurance Sampling Evaluation of Access and Coverage (SLEAC) Technical Reference. 2012. Available: http://www.fantaproject.org/sites/default/files/resources/SQUEAC-SLEAC-Technical-Reference-Oct2012_0.pdf

[13] Coverage Monitoring Network Profile Field Exchange, Emergency Nutrition Network. 2013;46, 43. Available: http://www.ennonline.net/pool/files/fex/fx-46-web.pdf

[14] Guerrero S, Myatt M, Collins S. Determinants of coverage in Community-based therapeutic Care programmes: towards a joint quantitative and qualitative analysis. Disasters. 2010; 34, 571–85. 10.1111/j.1467-7717.2009.01144.x 20002705

[15] Project Sphere, Sphere Handbook: Humanitarian Charter and Minimum Standards in Disaster Response. Oxford, Oxfam Publishing 2004 10.1111/j.0361-3666.2004.00245.x 20958782

[16] Collins S, Sadler K, Dent N, Khara T, Guerrero S, Myatt M *et al* Key issues in the success of community-based management of severe malnutrition: technical background paper for WHO consultation. 2005. Available: http://www.who.int/nutrition/topics/backgroundpapers_Key_issues.pdf 10.1177/15648265060273S304 17076213

[17] International Food Policy Research Institute. Global Nutrition Report 2014: Actions and Accountability to Accelerate the World’s Progress on Nutrition. Washington, DC. 2014 Available: http://www.ifpri.org/sites/default/files/publications/gnr14.pdf 10.3945/an.115.008599 PMC4424785 25979494

[18] Puett C, Hauenstein Swan S, Guerrero S. Access for All, Volume 2: What factors influence access to community-based treatment of severe acute malnutrition? Coverage Monitoring Network. 2013. Available: http://www.coverage-monitoring.org/wp-content/uploads/2013/12/AAH-Policy-Paper2-06-12-13-updated.pdf

[19] Schofield L, Gizaw Lalcha S, Getachew T. SQUEAC in routine monitoring of CMAM programme coverage in Ethiopia. Field Exchange, Emergency Nutrition Network. 2010;38: 35.

[20] Guevarra E, Guerrero S, Myatt M. Using SLEAC as a wide-area survey method. Field Exchange, 2012;42, 39–44. Available: http://fex.ennonline.net/42/using

[21] Perez Bernabe B. Semi-Quantitative Evaluation of Access and Coverage in Huambo, Angola. Coverage Monitoring Network. 2013. Available: http://www.coverage-monitoring.org/wp-content/uploads/2013/09/SQUEAC-Report_Huambo-ANG_VF-26-07.pdf

[22] Myatt M, Guerrero S. Why coverage is important: efficacy, effectiveness, coverage and the impact of CMAM interventions. Field Exchange. 2013; 45, 39–41. Available at http://fex.ennonline.net/45/coverage

[23] Tekeste A, Wondafrash M, Azene G, Deribe K. Cost effectiveness of community-based and in-patient therapeutic feeding programs to treat severe acute malnutrition in Ethiopia. Cost Eff Resour Alloc. 2012;10:4 10.1186/1478-7547-10-4 22429892 PMC3323427

[24] Sadler K, Puett C, Mothabbir G, Myatt M. Community Case Management of Severe Acute Malnutrition in Southern Bangladesh: A partnership study between the Feinstein International Center, Tufts University, Save the Children USA, The Bangladesh Institute of Public Health Nutrition, Sher-E- Bangla Medical College & Hospital, and the Bangladesh Director General of Health Services, Feinstein International Center, Medford MA 2011.

